# Solar-powered oxygen delivery: study protocol for a randomized controlled trial

**DOI:** 10.1186/s13063-015-0814-y

**Published:** 2015-07-09

**Authors:** Saleh Nyende, Andrea Conroy, Robert Opika Opoka, Sophie Namasopo, Kevin C. Kain, Arthur Mpimbaza, Ravi Bhargava, Michael Hawkes

**Affiliations:** Department of Paediatrics, Jinja Regional Referral Hospital, Jinja, Uganda; Department of Medicine, University of Toronto, Toronto, Canada; Department of Paediatrics and Child Health, Mulago Hospital and Makerere University, Kampala, Uganda; Institute of Medical Sciences, University of Toronto, Toronto, Canada; Sandra A. Rotman Laboratories, McLaughlin-Rotman Centre for Global Health, Toronto, Canada; McLaughlin Centre for Molecular Medicine, Toronto, Canada; Tropical Disease Unit, Toronto General Hospital, Toronto, Canada; Infectious Disease Research Collaboration, Kampala, Uganda; Department of Diagnostic Imaging, University of Alberta, Edmonton, Canada; Division of Infectious Diseases, Department of Pediatrics, University of Alberta, Edmonton, Canada

**Keywords:** Oxygen, Pneumonia, Pediatrics, Global health, Resource-limited hospital

## Abstract

**Background:**

Pneumonia is a leading cause of childhood mortality globally. Oxygen therapy improves survival in children with pneumonia, yet its availability remains limited in many resource-constrained settings where most deaths occur. Solar-powered oxygen delivery could be a sustainable method to improve oxygen delivery in remote areas with restricted access to a supply chain of compressed oxygen cylinders and reliable electrical power.

**Methods/Design:**

This study is a randomized controlled trial (RCT). Solar-powered oxygen delivery systems will be compared to a conventional method (oxygen from cylinders) in patients with hypoxemic respiratory illness. Enrollment will occur at two sites in Uganda: Jinja Regional Referral Hospital and Kambuga District Hospital. The primary outcome will be the length of hospital stay. Secondary study endpoints will be mortality, duration of supplemental oxygen therapy (time to wean oxygen), proportion of patients successfully oxygenated, delivery system failure, cost, system maintenance and convenience.

**Discussion:**

The RCT will provide useful data on the feasibility and noninferiority of solar-powered oxygen delivery. This technological innovation uses freely available inputs, the sun and the air, to oxygenate children with pneumonia, and can be applied “off the grid” in remote and/or resource-constrained settings where most pneumonia deaths occur. If proven successful, solar-powered oxygen delivery systems could be scaled up and widely implemented for impact on global child mortality.

**Trial registration:**

Clinicaltrials.gov registration number NCT0210086 (date of registration: 27 March, 2014)

## Background

Pneumonia is a leading cause of pediatric mortality globally, causing 0.9 million deaths/year [1]. Countries in Africa and Asia report two to 10 times more cases of pneumonia than industrialized countries like the USA, and the majority of pneumonia deaths [2–4]. Respiratory distress and hypoxemia are presenting features of life-threatening bacterial pneumonia, as well as pulmonary tuberculosis, sepsis and severe malaria. These common and treatable infections lead to hypoxemia as a final common pathway, for which oxygen (O_2_) therapy is an essential supportive therapy.

Large gaps remain in the case management of children presenting to African hospitals with respiratory distress, including supplemental oxygen. A 2012 survey found that only 44 % of 231 health centers, district hospitals, and provincial/general hospitals in 12 African countries had access to oxygen on a continuous basis [5]. In Malawi in 2008, oxygen was not available in four out of five district hospital pediatric wards visited, and health workers did not know when or how to administer oxygen to children [6]. Similarly, in another survey, oxygen was not available for 13 % of hypoxemic children admitted to five hospitals in New Guinea [7]. However, improved oxygen delivery systems can lead to measurable improvements in survival. A multihospital effectiveness study in Papua New Guinea demonstrated a reduction in mortality from childhood pneumonia from 5.0 % to 3.2 % (35 % reduction in mortality) after implementation of an enhanced oxygen delivery system (pulse oximetry, oxygen concentrators and training) [8].

Current methods commonly used to deliver hospital oxygen in resource-limited settings include compressed oxygen cylinders and oxygen concentrators. Cylinders require a reliable supply chain linking the oxygen production plant to the hospital, which may be compromised by poor road conditions, costs of transportation and weak stock management. Furthermore, while cylinders themselves are robust, tank regulators, used to deliver a constant and metered flow of oxygen, are frequently ill fitting and poorly maintained, resulting in substantial gas leak and waste of compressed oxygen. Oxygen concentrators represent a significant technological advance, purifying oxygen from ambient air through selective adsorption of nitrogen using aluminum silicate sieve beds. However, these require a constant uninterrupted electrical supply, which may not be available in resource-poor settings. We propose a novel strategy for oxygen delivery that could be implemented in remote locations with minimal access to oxygen cylinders or an electrical power supply: solar-powered oxygen (SPO2) delivery. Here, we describe the protocol for a randomized controlled trial (RCT) to compare SPO2 to conventional oxygen delivery from cylinders.

## Methods/Design

### Study design

**Fig. 1:**
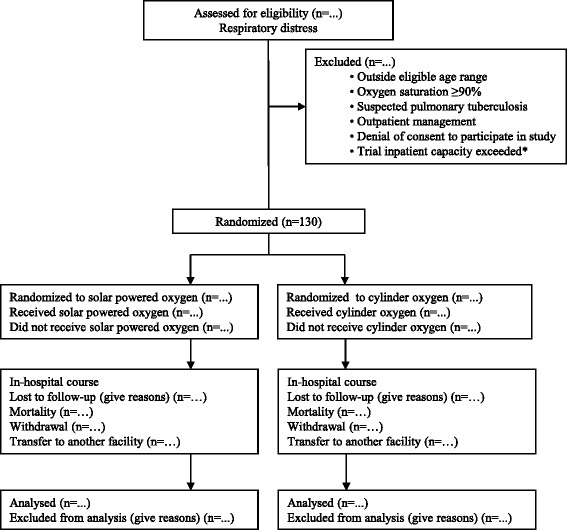
Trial flow diagram. *The trial capacity is two beds with a concentrator and two beds with a cylinder. In case of two patients already enrolled in the same arm, subsequent patients were not enrolled because of the potential inability to randomize to the occupied beds

The study is a prospective, randomized, controlled clinical trial comparing solar-powered oxygen delivery to oxygen in compressed cylinders in children under 13 years of age admitted with hypoxemic respiratory illness. The patient flow diagram is shown in Fig. 1.

### Study objectives

The overall objective is to demonstrate the noninferiority of solar-powered oxygen delivery relative to standard oxygen delivery. Specifically, we will compare the length of stay in children hospitalized with acute hypoxic respiratory illness randomized to SPO2 versus cylinder oxygen. The working hypothesis is that the length of stay is not increased in children treated with SPO2 compared to children receiving cylinder oxygen. Secondary outcomes will be compared between groups, including patient-specific outcomes (mortality, duration of supplemental oxygen therapy (time to wean O_2_), proportion of patients successfully oxygenated, and clinical severity scores) and oxygen delivery system indicators (system failures, cost, maintenance and convenience of use).

### Eligibility criteria

#### Inclusion criteria

Inclusion criteria include the following:

1. Age <13 years
2. Integrated Management of Childhood Illness (IMCI) defined severe pneumonia or very severe disease [9]
3. Hypoxemia (peripheral saturation<90 %) based on noninvasive pulse oximetry
4. Hospital admission warranted based on clinician judgment
5. Consent to blood sampling and data collection

#### Exclusion criteria

Exclusion criteria include the following:

1. Outside eligible age range
2. Peripheral oxygen saturation ≥90 %
3. Suspected pulmonary tuberculosis
4. Outpatient management
5. Denial of consent to participate in study
6. Trial inpatient capacity exceeded

The reason for exclusion of patients with pulmonary tuberculosis is for infection control, in order to avoid the risk of transmitting tuberculosis to other patients housed in the study unit. The trial inpatient capacity at each study site is two beds with a concentrator and two beds with a cylinder. In cases where two patients are already enrolled in the same arm, subsequent patients will not be enrolled because of the potential inability to randomize to the occupied beds.

### Study setting

The trial will take place at two hospitals in Uganda: (1) the Jinja Regional Referral Hospital is located in in mid-eastern Uganda, which serves a large catchment area in the Busoga Region, and (2) Kambuga Hospital in Western Uganda, a remote site with limited road access. Both are severely resource-limited hospitals with inconsistent electrical power supply, and both were using oxygen from cylinders and concentrators at the time of study planning. Uganda has a high burden of child mortality and pneumonia: the under-five mortality rate in 2008 was 135 deaths per 1000 (19^th^ highest rate worldwide), and pneumonia is the fourth leading cause of mortality in Ugandan children under five (11 %) [1].

### Treatment groups

Children will be randomly assigned to receive oxygen using either SPO2 or cylinder oxygen in a 1:1 ratio.

The intervention arm is oxygen from a solar-powered concentrator. The SPO2 systems, which are built in Jinja and Kambuga, consist of photoelectric panels (80 W or 175 W panels, Solarworld, Freiburg, Germany), charge controller (FLEXmax 80, OutBack Power, Arlington, WA, USA or 40 A/24 V Controller, Steca Elektronik GmbH, Memmingen, Germany), bank of gel batteries (220 Ah/12 V UltraPower gel batteries, Victron Energy BV, Almere Haven, The Netherlands), power inverter (3000 VA/48 V Inverter/Charger or 1600 VA/24 V Inverter/Charger, Victron Energy BV, Almere Haven, The Netherlands) and oxygen concentrator (model 525 KS, DeVilbiss Healthcare LLC, Somerset, PA, USA). The system is designed to run independently of hydroelectric power source (exclusively driven by solar energy), and provide continuous uninterrupted oxygen (up to 5 L/min) throughout both day and night (using energy stored in battery bank). The total cost of each system was approximately US $18,000.

The control arm consists of oxygen in a cylinder with flow regulator, tubing and nasal prongs or mask for delivery to the patient. Cylinders (pressure, capacity) can be purchased from commercial suppliers in Uganda (Oxygas, Kampala, Uganda) at a cost of US $200 and are refillable at a cost of US $14. In addition, a regulator (US $200) is needed. On average, one pediatric patient with pneumonia will consume approximately 1-6 cylinders of oxygen during the course of hospitalization for pneumonia. In order to have a reliable supply of oxygen, given procurement and transport times, we estimate that six cylinders need to be stocked on the hospital premises.

### Randomization method

Block randomization by study site will be employed, using a computer-generated list. Treatment allocation will be kept in sequentially numbered, sealed, opaque envelopes, which will be drawn for each randomized participant. We will retain all envelopes and records for quality-monitoring purposes.

### Outcome measures

Length of stay will be the primary outcome. A standardized and objective decision rule will be used for hospital discharge. All of the following criteria will be required for discharge: (1) off oxygen for at least 24 hours (requires saturation ≥90 % on room air); (2) absence of fever for 24 hours; (3) vital signs stable; and (4) drinking and/or breastfeeding well.

Several secondary outcomes will be recorded. Patient-specific outcomes will be measured in each group: mortality, proportion of patients successfully oxygenated, duration of oxygen therapy, and clinical severity score. The proportion of patients successfully oxygenated will be defined as the number of patients who achieve a saturation ≥90 % for ≥12 hours after initiating oxygen therapy. The clinical severity scores used will be the previously published and validated Lambaréné Organ Dysfunction Score (LODS) [10] and the Respiratory Index of Severity in Children (RISC) [11].

Oxygen delivery system variables will include direct costs (capital investment, operating costs, system maintenance, *etcetera*), details of maintenance needs, convenience of use of the systems and proportion of patients for whom the delivery system failed. System failure will be defined as inadequate oxygen production to meet patient needs for any reason. Examples would include battery failure for SPO2 or out-of-stock cylinder oxygen.

### Safety

Oxygen therapy is known to be a life-saving intervention, and this study will improve hospital capacity for the treatment of hypoxemic children. While patients will be randomized to one of two possible oxygen delivery systems, there are no risks, and benefits include potentially life-saving access to oxygen, regardless of delivery modality. Study participants will be managed at the Jinja Regional Referral Hospital according to national guidelines and local clinical practices. Outcome assessment in the study will not pose any risk to the study participants.

Adverse events, defined according to Good Clinical Practice (GCP) guidelines as any untoward event occurring in a study participant, will be logged prospectively and tabulated at the end of the study, disaggregated by study arm. Severe adverse events, defined as those adverse events that prolong hospitalization or lead to permanent disability or death, will be reported to regulatory authorities in an expedited manner and reported separately in the safety analysis.

This study involves hypoxemic patients with severe pneumonia or very severe disease, and the mortality is expected to be high because of the underlying illness. Mortality among patients receiving SPO2 will to be interpreted in comparison to patients receiving O_2_ by standard method (cylinders).

### Ethical considerations

The Makerere University School of Biomedical Sciences Research and Ethics Committee (SBS-REC, # SBS 139), together with the University Health Network Research Ethics Board (UHN REB, # 13-6168-AE) of the University of Toronto have reviewed and approved the study protocol. The Uganda National Council on Science and Technology (UNCST, # SS 331) has also approved the study.

Written informed consent will be obtained from the caregivers of all children that will participate in the study. The consent has been translated into local languages (Luganda, Lusoga and Rukiiga). Illiteracy is common in the study area, requiring adaptation of the consent process. For those who do not know how to read and write, an independent witness will be present during the informed consent process and will sign the consent form as a witness. The parent themselves will affix a thumbprint to the consent form. Assent will be solicited for patients above 8 years of age.

### Sample size calculations

We will enroll a total of 130 patients. This sample size was calculated as follows. A 1:1 ratio of patients will be assigned to SPO2 or control (cylinder O_2_). We consider a 1-day prolongation of hospital stay to be clinically significant (noninferiority margin). Using pilot data from a prospective study at Jinja Regional Referral Hospital, 69 patients with hypoxic pneumonia had a mean (standard deviation) length of stay of 2.6 (2.1) days (unpublished data). By standard calculations for normally distributed data, 57 patients in each group will provide 80 % power to demonstrate noninferiority of SPO2 relative to oxygen by cylinder at α = 0.05 (one-sided). This sample size was increased by 15 % to account for a probable non-Gaussian distribution of the data [12].

### Primary analysis

Length of stay (in days) will be coded as a whole number (continuous variable). Difference in length of stay between groups will be compared using the nonparametric method of Hodges and Lehman. If the *a priori* noninferiority margin (Δ = 1 day) is below the lower limit of the 95 % confidence interval for the median of differences, the trial will conclude that SPO2 is noninferior to cylinder oxygen. In order to account for all patients randomized (“intention-to-treat” analysis), we will perform sensitivity analyses to account for missing data due to mortality, study withdrawal, and transfer to another facility. A *per protocol* population (non-fatal cases discharged home from hospital) will be used to assess the robustness of the primary result (non-inferiority). Details of the sensitivity analyses will be included in a Statistical Analysis Plan, which will be closed as early as possible in the conduct of the trial.

### Secondary outcomes

The mortality will be compared between groups using the chi-squared statistic, or Fisher’s exact test, as appropriate. The proportion of patients successfully oxygenated and proportion of patients for whom the oxygen delivery system failed will be analyzed in a similar manner. The duration of oxygen therapy will be compared using nonparametric methods (Mann-Whitney U-test) and/or Cox-proportional hazard models. Costs will be carefully tabulated (capital investment, ongoing costs, and system maintenance, *etcetera*) and summarized for each group of patients. Details of maintenance needs and convenience of use of the systems will be documented and tabulated. The LODS and RISC (clinical severity scores) will be assessed and recorded serially over the course of hospital admission; comparison between groups will make use of linear mixed effects models to compare the longitudinal time course of the clinical severity score during convalescence.

## Discussion

Our protocol describes a randomized controlled trial to test the hypothesis that solar-powered oxygen delivery is noninferior to standard methods (oxygen from cylinders) for the supportive care of children with hypoxemia and respiratory distress. Demonstrating the utility of this technological innovation will represent an important step toward scale-up and implementation of reliable oxygen delivery in remote and resource-limited hospitals.

The principle of the solar-powered oxygen delivery system relies on two existing technologies, photoelectric panels and oxygen concentrator, to yield a stream of oxygen that requires only sun and air as inputs. Existing oxygen concentrators use pressurized beds of aluminum silicate to adsorb nitrogen from ambient air, generating relatively pure oxygen (up to 95 %). Using solar energy to power the concentrator allows it to operate in settings where the supply of electricity is unreliable. Previous studies have described the clinical use of oxygen concentrators in developing countries [6, 13–15], but, to our knowledge, only one narrative report to date from the Gambia has described a solar-powered oxygen system (design and cost), and clinical data were not reported [16].

Noteworthy aspects of this trial include its setting in two severely resource-limited hospitals, where hypoxemic patients do not have reliable access to oxygen, intensive care environment, endotracheal intubation, or mechanical ventilatory support. This situation is common across hospitals in sub-Saharan Africa [5], and contributes to elevated mortality rates from pediatric pneumonia. Threats to consistent oxygen supply in our centers and others include weak supply chain for oxygen cylinders, inconsistent electric power supply, and lack of well-maintained oxygen concentrators. The use of solar-powered oxygen eliminates the need for oxygen cylinders and electricity but will still require minimal equipment maintenance. The study setting in equatorial Uganda is also representative of many African sites, where solar energy is abundant and can be harnessed for oxygen purification from ambient air. Our trial will provide “real-life” data on system operation under field conditions including variability in sun intensity throughout the day and with weather conditions (energy available) and patient oxygen demands. These features increase the generalizability of our findings to similar settings and will inform future refinements and optimization of the solar-powered oxygen delivery system.

One limitation of this study is the lack of blinding. Given the nature of the intervention (oxygen concentrator or cylinder), it is not feasible to blind clinicians or participants to the intervention in our setting. Decisions about the primary outcome (length of stay) are subject to clinician judgment, opening the possibility of bias. To mitigate this potential bias, we will use objective criteria for hospital discharge, which will be applied uniformly to patients in both arms. We have chosen length of stay (continuous variable), rather than mortality as the primary study endpoint for pragmatic reasons. The binary outcome mortality would require a much larger and costlier study to show noninferiority (sample size of n = 1,134 per group needed to show noninferiority within a margin of 5 % absolute risk difference). For the sample size calculation, we used a one-sided 5 % significance level, rather than the more conventional two-sided approach. This reduced the total sample size by approximately 30 patients while still permitting demonstration of noninferiority with 95 % confidence (based on the lower limit of the one-sided confidence interval).

Several potential confounding factors may impact our primary outcome (length of stay) or secondary outcomes (for example, mortality) among patients with hypoxemia. These include host factors (for example, patient age, underlying immune deficiency like HIV), disease-specific factors (for example, pathogen and severity of infection), and co-treatments (for example, antibiotics). Our randomized design is expected to distribute these variables equally between study arms, and we have not attempted to stratify the cohort due to the modest sample size. Secondary analyses (for example, multivariable regression) may be undertaken to explore the important predictors of patient outcomes in this cohort.

Potential impacts of solar-powered oxygen delivery include a reduction in pneumonia-specific and overall child mortality if this technology can be brought to scale and implemented widely in Africa and Asia. Toward this goal, our RCT will provide strong empirical data to support the feasibility and noninferiority of SPO2 in resource-limited African hospitals. We anticipate that this trial will also provide valuable data for future design and refinement of solar-powered oxygen delivery systems, including the potential for commercial development. A significant reduction in the number of children dying of pneumonia may be realized with this pragmatic method to provide supportive therapy with oxygen globally.

## Trial status

Recruitment of study participants ongoing.

## Abbreviations

LODS: Lambaréné Organ Dysfunction Score
REB: Research Ethics Board
RISC: Respiratory Index of Severity in Children
SBSREC: School of Biomedical Sciences Research and Ethics Committee
SPO2: solar-powered oxygen
UNCST: Uganda National Council on Science and Technology
